# Outcome of catheter ablation of non-reentrant ventricular arrhythmias in patients with and without structural heart disease

**DOI:** 10.1186/s40001-020-0400-y

**Published:** 2020-03-17

**Authors:** Ruben Schleberger, Mario Jularic, Tim Salzbrunn, Claudia Hacke, Jana M. Schwarzl, Boris A. Hoffmann, Daniel Steven, Stephan Willems, Marc D. Lemoine, Christian Meyer

**Affiliations:** 1 Department of Cardiology-Electrophysiology, University Heart and Vascular Center, University Medical Center Hamburg-Eppendorf, Martinistr. 52, 20246 Hamburg, Germany; 2 Department of Cardiology, Asklepios Klinik St. Georg, Lohmühlenstr. 5, 20099 Hamburg, Germany; 3 Institute of Medical Biometry and Epidemiology, University Medical Center Hamburg-Eppendorf, Martinistr. 52, 20246 Hamburg, Germany; 4 Department of Electrophysiology, Asklepios Klinikum Harburg, Eißendorfer Pferdeweg 52, 21075 Hamburg, Germany; 5 Department of Electrophysiology, University Heart Center Cologne, University Hospital Cologne, Kerpener Str. 52, 50937 Cologne, Germany; 6 DZHK (German Centre for Cardiovascular Research), Partner Site Hamburg/Kiel/Lübeck, Hamburg, Germany

**Keywords:** Idiopathic ventricular tachycardia, Non-reentrant ventricular tachycardia, Structural heart disease, Ventricular arrhythmia, VT ablation

## Abstract

**Background:**

Catheter ablation of non-reentrant, commonly termed “idiopathic” ventricular arrhythmias (VA) is highly effective in patients without structural heart disease (SHD). Meanwhile, the outcome of catheter ablation of these arrhythmias in patients with SHD remains unclear. This study sought to characterize the outcome of patients with and without SHD undergoing catheter ablation of non-reentrant VA.

**Methods:**

In this single-centre study the acute and long-term outcome of 266 consecutive patients undergoing catheter ablation of non-reentrant VA was investigated. In 41.0% of patients a SHD was present (*n* = 109, 80.7% male, age 59.1 ± 14.7 years), 59.0% had no SHD (*n* = 157; 44.0% male, age 49.9 ± 16.5 years).

**Results:**

Acute procedural success (absence of spontaneous or provoked VA at the end of procedure and within 48 h after the procedure) was achieved in 89.9% of patients with SHD vs. 94.3% without SHD (*p* = 0.238). During a mean follow-up of 34.7 ± 15.1 months a repeat catheter ablation was performed in 19.6% of patients with SHD vs. 13.0% without SHD (*p* = 0.179). Patients with dilated cardiomyopathy (DCM) were the most likely to require a repeat ablation procedure (32.0% of patients with DCM vs. 13.0% without SHD; *p* = 0.022). Periprocedural complications occurred in 5.5% of patients with SHD vs. 5.7% without SHD (*p* > 0.999). All complications were managed without sequelae.

**Conclusions:**

The outcome of catheter ablation of non-reentrant VA in patients with SHD appears good and is comparable to patients without SHD. A slightly higher rate of repeat ablations was observed in patients with DCM.

## Background

Catheter ablation of non-reentrant ventricular arrhythmias (VA) has been shown to be highly effective in the absence of structural heart disease (SHD) [1]. These VAs, commonly termed “idiopathic”, originate frequently from the right and left ventricular outflow tracts, as well as aortic cusps and surrounding tissue including mitral annulus and papillary muscles [2, 3]. They are generally accepted to be benign although rare fatal outcomes are reported [4, 5]. By current international guidelines, catheter ablation in patients without SHD has a class I indication for VA originating from the outflow tracts [6]. While catheter ablation of non-reentrant VA is now well established in many electrophysiological laboratories, patients with an underlying SHD have been excluded from most studies addressing non-reentrant VA. Studies investigating outcome in patients with SHD are mainly restricted to reentrant VA related to an abnormal myocardial substrate. However, the prevalence of non-reentrant VA unrelated to abnormal myocardial substrate in patients with SHD presenting for catheter ablation has been found to be > 20%, while data on long-term outcome in these patients are sparse [7]. Therefore, we here present the short- and long-term outcome of catheter ablation of non-reentrant VA in a relatively large cohort of patients with SHD.

## Methods

### Patient selection

Case records from the University Heart and Vascular Center database were reviewed [8]. Between October 2012 and December 2015 387 patients with VA (ventricular tachycardia (VT) and/or premature ventricular contractions (PVC)) were referred to our institution for a catheter ablation. The present analysis includes all patients (*n* = 266) with non-reentrant VA originating from areas of structurally normal myocardium. Patients with and without SHD were included. Non-reentrant VA were defined as arrhythmias of presumably focal origin. They were distinguished from reentrant VA according to their mode of initiation, appearance in activation and entrainment mapping, their local electrogram characteristics (e.g. unipolar signal with QS pattern) and their response to overdrive pacing [7]. A definitive arrhythmia mechanism could not be defined for all VAs, though only patients with suspected focal mechanism were included into the study. Patients with evidence for a VA with reentry mechanism or non-reentrant VA originating from areas of structurally abnormal myocardium were excluded from further analysis.

### Definition of structural heart disease

All patients were screened for SHD using echocardiography, cardiac magnetic resonance imaging and/or myocardial scintigraphy. SHD was defined according to the classification of cardiomyopathies in line with the current guidelines of the European Society of Cardiology [9, 10]. Pathologic findings in the diagnostic workup including wall motion abnormalities, inhomogenicity of the myocardium, late gadolinium enhancement, perfusion defects, pathological Q-waves, and low-voltage areas in voltage mapping (> 1 cm^2^ with a voltage < 1,5 mV) [11] were counted as a SHD [7]. Reduced left ventricular ejection fraction (LVEF) without other cardiac abnormalities that normalized after ablation (LVEF < 50% with improvement to > 50%) was defined as an arrhythmia induced cardiomyopathy and not counted as SHD [12].

### Irrigated radiofrequency ablation

All patients underwent conscious sedation under spontaneous ventilation and continuous monitoring of oxygen saturation and blood pressure [13]. All antiarrhythmic drugs with exception of amiodarone had been discontinued for at least five half-lives. Access to the mapping region was gained through cannulation of the femoral vein or artery. In all patients cardiac anatomy was displayed using a three-dimensional electroanatomical mapping system (Carto^®^ 3 System, Biosense Webster Johnson and Johnson, Diamond Bar, CA, USA). Activation mapping and entrainment mapping were performed if possible. Whenever considered helpful, pace mapping was performed for identification of the PVC or VT origin site using the distal bipolar electrode of the mapping catheter at a pacing cycle length of 500 ms with the lowest stimulus amplitude (varying from 3 to 10 mA) and pulse width (1.0–2.0 ms) producing stable ventricular capture [8, 13]. If no VA could be registered, programmed stimulation was performed with two different basic cycle lengths (510 and 440 ms) and up to three extra stimuli with a minimal coupling interval of 180 ms. In all cases incremental atrial and ventricular pacing was performed following the programmed stimulation. The induced VT was defined as the clinical VT when cycle length (within 20 ms) and morphology matched previous recordings. In the case of non-inducibility, the stimulation protocol was repeated during intravenous orciprenaline infusion (5 mg/500 mL NaCl 0.9%) with at least 20% increment of heart rate [14–16]. If the clinical VA could still not be induced, the patient was excluded from further analysis.

Ablations were performed using a 3.5-mm externally irrigated-tip ablation catheter (NaviStar ThermoCoolVR, Biosense Webster) after identification of the ablation site (earliest activation of at least − 20 ms before QRS-onset and/or perfect pace map). Radiofrequency applications were performed in a temperature-controlled mode with a maximum temperature of 48 °C. The maximum output chosen was 20–40 W depending on the location. When an acceleration or reduction of the cycle length of the VT or the frequency of the PVC was observed during the first 20 s of the application, the radio frequency energy delivery was continued for a maximum of 180 s. Otherwise the radio frequency delivery was terminated and the catheter was repositioned [8, 13].

### Procedure success and follow-up

Acute ablation success was defined as absence of spontaneous or provoked clinical VA at the end of the procedure, and absence of the targeted VA on 48-h electrocardiography (ECG) monitoring after ablation. VA burden was documented on Holter monitoring before and after ablation. Major complications were defined as complications leading to a prolonged hospital stay, permanent inconveniences or surgery. The follow-up was performed in our outpatient clinic or for some patients at the referring physician’s office. Patients with insufficient follow-up data (*n* = 10) were excluded from the follow-up analysis.

### Statistics

Continuous variables are expressed as mean ± standard deviation. Categorical variables are presented as frequencies and percentages. Independent samples t-tests were carried out to assess differences between patients with and without SHD in all continuous variables. Categorical variables were compared using Fisher’s exact tests. We tested for significant mean change within each group using paired *t*-tests and compared mean change between groups by independent *t*-tests. Survival curves depicting the time to a repeat ablation procedure were estimated for each group, considered separately, using the Kaplan–Meier method and compared statistically using the log rank test. All of the models present available case analysis. A two-tailed *p *<0.05 was considered statistically significant. Statistical analyses were performed using the software GraphPad Prism 7.0 (GraphPad Software Inc., San Diego, CA, USA).

## Results

### Patient characteristics

266 consecutive patients with non-reentrant, idiopathic VA were included into the study (Table 1). In 41.0% of these patients (109 out of 266) a SHD was present, 59.0% (157 out of 266) had no SHD. Ischemic heart disease (ICM) was the most common type of SHD with 42.2% (46 out of 109), followed by dilated cardiomyopathy (DCM) with 25.7% (28 out of 109). The remaining 35 patients had previous myocarditis (11.0%; 12 out of 109), hypertrophic cardiomyopathy (HCM; 10.1%; 11 out of 109) or other rare cardiomyopathies (11.0%; 12 out of 109).

**Table 1:** Patient characteristics

	Total (*n* = 266)	SHD (*n* = 109)	No SHD (*n* = 157)	*p* value (SHD vs. no SHD)
Male gender	157 (59)	88 (80.7)	69 (44.0)	< 0.001*
Age, years	53.7 ± 16.4	59.1 ± 14.7	49.9 ± 16.5	< 0.001*
Body mass index, kg/m^2^	26.6 ± 4.8	27.1 ± 4.3	26.3 ± 5.1	0.139
LVEF, %	56 ± 10	49 ± 11	60 ± 6	< 0.001*
ICD	30 (11.3)	28 (25.7)	2 (1.3)	< 0.001*
Hypertension	122 (45.9)	68 (62.4)	54 (34.4)	< 0.001*
Diabetes	19 (7.1)	13 (11.9)	6 (3.8)	0.015*
Arrhythmias				
VT	31 (11.7)	21 (19.3)	10 (6.4)	0.002*
VT + PVC	42 (15.8)	20 (18.3)	22 (14.0)	0.396
PVC	193 (72.6)	68 (62.4)	125 (79.6)	0.003*
Acute success	246 (92.5)	98 (89.9)	148 (94.3)	0.238

*LVEF* left ventricular ejection fraction, *ICD* implantable cardioverter–defibrillator, *PVC* premature ventricular contraction, *SHD* structural heart disease, *VT* ventricular tachycardia

Values are *n* (%) or mean ± standard deviation. *p <0.05 was considered statistically significant

### Procedural success

Acute procedural success was achieved in 89.9% (98 out of 109) of patients with SHD vs. 94.3% (148 out of 157) of patients without SHD (*p* = 0.238). 72.6% (193 out of 266) had documented PVC, 11.7% (31 out of 266) were diagnosed with VT only and 15.8% (42 out of 266) had both PVC and VT. The patients with SHD presented more often with VT (37.6% vs. 20.4%; *p* = 0.002). When analysing the origin of the VA we found a higher number of left-sided VA in patients with SHD: 66.0% vs. 46.5% (*p* = 0.002). The locations of successful ablation are shown in Table 2.

**Table 2:** Analysis of the origin of ventricular arrhythmias

	Total (*n* = 266)	SHD (*n* = 109)	No SHD (*n* = 157)	*p* value (SHD vs. no SHD)
RV origin	121 (45.5)	37 (33.9)	84 (53.5)	0.002*
RVOT	108 (40.6)	30 (27.5)	78 (49.7)	< 0.001*
RV (other)	13 (4.9)	7 (6.4)	6 (3.8)	0.392
LV origin	145 (54.5)	72 (66.0)	73 (46.5)	0.002*
LVOT	16 (6.0)	5 (4.6)	11 (7.0)	0.601
LCC/RCC/NCC	47 (17.7)	22 (20.2)	25 (15.9)	0.415
AMC	16 (6.0)	3 (2.8)	13 (8.3)	0.071
GCV	12 (4.5)	6 (5.5)	6 (3.8)	0.577
MVA	4 (1.5)	3 (2.8)	1 (0.6)	0.308
Papillary muscles	2 (0.8)	2 (1.8)	0	0.167
LV summit	2 (0.8)	1 (0.9)	1 (0.6)	> 0.999
LV septal	13 (4.9)	11 (10.1)	2 (1.3)	0.002*
LV inf./posterior	18 (6.8)	11 (10.1)	7 (4.5)	0.085
LV anterior	5 (1.9)	3 (2.8)	2 (1.3)	0.403
LV lateral	1(0.4)	1 (0.9)	0	0.410
LV (other)	9 (3.4)	4 (3.7)	5 (3.2)	> 0.999

*AMC* aortomitral continuity, *GCV* great cardiac vein, *LCC* left coronary cusp, *LV* left ventricle, *LVOT* left ventricular outflow tract, *MVA* mitral anulus, *NCC* non-coronary cusp, *RCC* right coronary cusp, *RV* right ventricle, *RVOT* right ventricular outflow tract, *SHD* structural heart disease

Values are *n* (%). *p <0.05 was considered statistically significant

A total of 15 periprocedural complications occurred in all 266 patients (5.6%). Ten of these were related to the vascular access, four were major complications (Table 3). All adverse events were managed without sequelae.

**Table 3:** Complications after catheter ablation

	Total (*n* = 266)	SHD (*n* = 109)	No SHD (*n* = 157)	*p* value (SHD vs. no SHD)
Total complications	15 (5.6)	6 (5.5)	9 (5.7)	> 0.999
Major complications	4 (1.5)	1 (0.9)	3 (1.9)	0.648
Complication type				
Arteriovenous fistula	4 (1.5)	3 (2.8)	1 (0.6)	
Groin hematoma	2 (0.8)	0	2 (1.2)	
Pseudoaneurysm	4 (1.5)	2 (1.8)	2 (1.2)	
Pericardial effusion	2 (0.8)	0	2 (1.2)	
Other	3 (1.1)	1 (0.9)	2 (1.2)	

Values are *n* (%)

*SHD* structural heart disease

### Follow-up

For 256 out of 266 patients complete follow-up data were available for analysis (SHD: *n* = 102, no SHD: *n* = 154). The mean follow-up duration was 34.7 ± 15.1 months and did not differ between patient groups (36.6 ± 15.2 months in patients with SHD vs. 33.4 ± 15.0 months in patients without SHD, Table 4). Eight patients (six with SHD) died unrelated to the procedure or to arrhythmic events during the follow-up period. The causes of death were cancer (two patients), suicide (one patient) and terminal heart failure (five patients).

**Table 4:** Follow-up after catheter ablation

	Total (*n* = 256)	SHD (*n* = 102)	No SHD (*n* = 154)	*p* value (SHD vs. no SHD)
Duration of follow-up, months	34.7 ± 15.1	36.6 ± 15.2	33.4 ± 15.0	0.101
Patients with repeat ablation of any VA, *n* (%)	47 (18.4)	23 (22.5)	24 (15.6)	0.189
Patients with repeat ablation of initial VA, *n* (%)	40 (15.6)	20 (19.6)	20 (13.0)	0.179
PVC burden at baseline, %	17.0 ± 13.4	16.9 ± 13.8	17.1 ± 13.2	0.901
PVC burden at follow-up, %	3.8 ± 7.8	5.5 ± 9.5	2.8 ± 6.4	0.005*
PVC count at baseline, *n*	17,324 ± 14,918	16,208 ± 14,568	17,979 ± 15,147	0.309
PVC count at follow-up, *n*	3620 ± 7327	5154 ± 8617	2720 ± 6322	0.002*
LVEF at baseline, %	56 ± 10	49 ± 11	60 ± 6	< 0.001*
LVEF at follow-up, %	56 ± 10	50 ± 12	60 ± 5	< 0.001*
AAD at baseline, *n* (%)	150 (59.0)	80 (78.4)	70 (45.5)	< 0.001*
AAD at follow-up, *n* (%)	142 (55.5)	82 (80.4)	60 (39.0)	< 0.001*
Time to repeat ablation, months	11.5 ± 11.3	11.3 ± 9.8	11.8 ± 12.4	0.892

*AAD* antiarrhythmic drugs (class I–IV), *ECG* electrocardiogram, *LVEF* left ventricular ejection fraction, *PVC* premature ventricular contraction, *SHD* structural heart disease, *VA* ventricular arrhythmia

Values are mean ± standard deviation or *n* (%). *p <0.05 was considered statistically significant. Patients with completed follow-up (*n* = 256 out of 266)

**Fig. 1:**
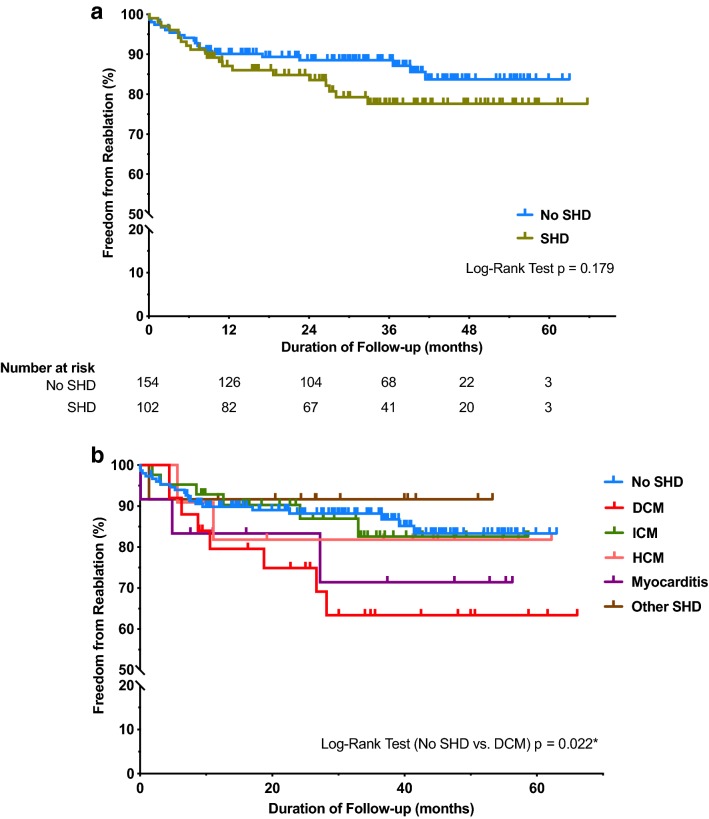
Freedom from repeat ablations. **a** Patients with structural heart disease vs. patients without structural heart disease. The rate of repeat ablations of the initially ablated arrhythmia did not differ significantly between patients with and without structural heart disease. Patients are censored at the end of their follow-up period. *SHD* patients with structural heart disease. **b** Subgroups of patients with structural heart disease vs. Patients without structural heart disease. Patients with dilated cardiomyopathy had a higher repeat ablation rate than patients without structural heart disease. The other subgroups did not differ significantly. Patients are censored at the end of their follow-up period. *p < 0.05 was considered statistically significant. *DCM* dilated cardiomyopathy, *HCM* hypertrophic cardiomyopathy, *ICM* ischemic cardiomyopathy, *SHD* patients with structural heart disease

During the follow-up period 22.5% (23 out of 102) of patients with SHD and 15.6% (24 out of 154) of patients without SHD required a repeat ablation of VA (*p* = 0.189; Fig. 1a; Table 4). Specifically, 20 patients with SHD (19.6%) vs. 20 patients (13.0%) without SHD had a repeat ablation of the same recurring arrhythmia from the initial ablation (*p* = 0.179). In another three patients treatment with sodium channel blockers and in seven patients with amiodarone was initiated due to VA.

An analysis of subgroups showed that the reablation rate of VA with origin in the right ventricle and VA originating from the left ventricle did not differ (12.9% vs. 17.9%, *p* = 0.304). Patients with DCM had the highest rate of repeat ablation procedures (32.0%), which was significantly higher than patients without SHD (*p* = 0.022; Table 5). The rate of repeat ablation procedures of the other subgroups (ICM, HCM, myocarditis, other cardiomyopathies) did not differ from patients without SHD (Fig. 1b). 42.9% of the VA in DCM patients originated from the right ventricular outflow tract, in comparison to 22.2% in all other SHD patients. The rate of repeat ablation procedures of VA from the right ventricular outflow tract was 27.6% in patients with SHD (27.0% in DCM) and 9.2% in patients without SHD (*p* = 0.027).

**Table 5:** Repeat ablation procedures in patient subgroups

Subgroup (total *n* = 256)	Patients with repeat ablation (*n* = 40)	*p* value (SHD subgroup vs. no SHD)
Patients with ICM (*n* = 42)	6 (14.3)	0.801
Patients with DCM (*n* = 25)	8 (32.0)	0.022*
Patients with HCM (*n* = 11)	2 (18.2)	0.643
Patients with myocarditis (*n* = 12)	3 (25)	0.220
Patients with other SHD (*n* = 12)	1 (8.3)	> 0.999
Patients without SHD (*n* = 154)	20 (13.0)	

*DCM* dilated cardiomyopathy, *HCM* hypertrophic cardiomyopathy, *ICM* ischemic cardiomyopathy, *SHD* structural heart disease, *VA* ventricular arrhythmia

Values are *n* (%). *p <0.05 was considered statistically significant

Patients with completed follow-up (*n* = 256 out of 266); repeat ablation procedures of the initially ablated arrhythmia were analysed

### PVC burden and left ventricular function

**Fig. 2:**
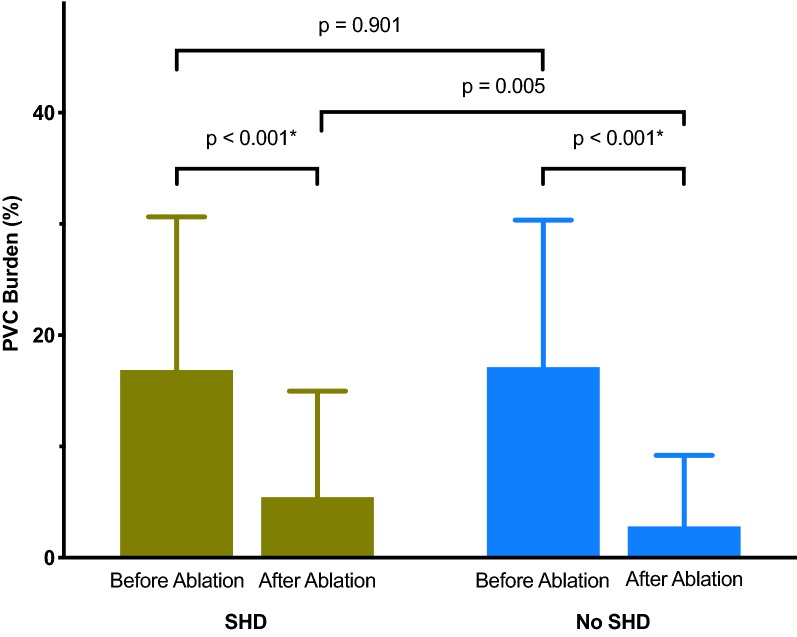
The impact of catheter ablation on the burden of premature ventricular contractions. A postprocedural reduction of the burden of premature ventricular contractions was observed in patients with and without structural heart disease. The burden at follow-up was higher in patients with structural heart disease. The bars show mean with standard deviation. *p < 0.05 was considered statistically significant. *PVC* premature ventricular contraction, *SHD* patients with structural heart disease

The average PVC burden before ablation was 16.9 ± 13.8% in patients with SHD and 17.1 ± 13.2% in patients without SHD (*p* = 0.901). The postprocedural PVC burden significantly decreased in both groups (− 11,4 ± 15,5% vs. − 14,3 ± 13,4%), but remained higher in patients with SHD (5.5 ± 9.5% vs. 2.8 ± 6.4%; *p* = 0.005; Fig. 2).

In neither group a significant change in the LVEF was observed. Yet some individuals experienced substantial increases in LVEF. All patients showing a relevant improvement of their LVEF had a decrease in PVC burden to values below 2%.

## Discussion

The major findings of the present study are:

1. Symptomatic non-reentrant VA are common in patients with a wide variety of SHD.
2. Patients with SHD have a similar short- and long-term outcome following catheter ablation of non-reentrant VA to patients without SHD. The subgroup of patients with DCM has a higher rate of repeat ablations.
3. Patients with SHD have a slightly higher burden of PVC at follow-up.

### Prevalence of idiopathic ventricular arrhythmias in patients with SHD

The first studies on radiofrequency energy ablation of VA in the late 1980s and early 1990s elegantly paved the way for catheter-based treatment of affected patients with and without SHD [17, 18]. Meanwhile in patients with SHD the potential benefit of catheter ablation of reentrant VA has been established in multiple clinical studies [19]. Data on the treatment of non-reentrant VA in patients with SHD remain rare, since such patients have so far been excluded from most studies. However, the here reported analysis suggests that non-reentrant, so called “idiopathic” VA are more common in patients with SHD than previously described.

### Outcome of catheter ablation

The present findings demonstrate a comparable outcome to recent relatively large studies investigating non-reentrant, idiopathic VA [20]. Patients with an otherwise healthy myocardium and idiopathic VA are known to have recurrence rates with up to 22% repeat ablation procedures in a recent multicentre study with 20.4 months follow-up [1]. Our findings suggest that this holds true irrespective of the fact whether a SHD is present or not. Rates of repeat ablation procedures appear comparable for both, patients with and without SHD presenting with non-reentrant VA during our follow-up period of 34.7 ± 15.1 months.

SHD is often associated with several comorbidities including peripheral artery disease or chronic kidney disease. Those conditions are known to increase the risk of procedural complications [21]. A recent multicentre study including patients with different kinds of VA showed a risk of 11.9% for complications in patients with SHD and 4.4% for patients without SHD [22]. In contrast, in our collective comparing patients with non-reentrant VA the procedural safety was relatively high in both groups, patients with and without SHD, and comparable to other studies investigating patients without SHD.

### Repeat ablation procedures in different types of SHD

Catheter ablation of VA is well known to be challenging in some patients with SHD. This especially holds true in non-ischemic causes (46–61% recurrences in patients with DCM) [23]. Aptly our data demonstrate a higher rate of repeat ablation procedures in patients with DCM in comparison to patients without SHD. As DCM patients in the present study had a high percentage of VA from the right ventricular outflow tract—which generally had a good outcome—the higher repeat ablation rate does not seem to be caused by an anatomically hard-to-reach ablation target. An undetected substrate could have been responsible for the recurrences [24–26], though this seems unlikely as no suspicious late gadolinium enhancement was detected during magnetic resonance imaging in the right ventricular outflow tract of those patients. However, this cannot be completely ruled out. Another reason for more ablation procedures might be that there is evidence for a reduced efficacy of antiarrhythmic drugs in patients with DCM [27]. Further studies are needed to clarify this effect on the repeat ablation rate.

### Impact of ablation on PVC burden and left ventricular function

A high PVC count is known to be associated with congestive heart failure and increased mortality [28]. The impact of a reduction of the arrhythmia burden on the improvement of the LVEF after ablation is now becoming more evident [12]. We achieved an overall reduction in the mean PVC burden from 17.0 to 3.8% with a higher burden at follow-up in the SHD group. The reason for this might be the potentially more complex substrate or the SHD itself, leading to additional PVC from other locations.

Despite a relevant increase in LVEF in a few patients there was no effect of ablation on the average left ventricular function in both groups in the present study. There might be several factors contributing to the fact that we did not observe overall changes in LVEF, though in general an inconsistent improvement was also observed in other studies [29]. First, patients in both groups had a relatively good LVEF prior to ablation. Second, the natural deterioration in LVEF due to heart failure and the underlying myocardial substrate might outweigh the effects of the ablation for some patients, especially with a longer follow-up time.

## Conclusions

The short- and long-term outcome of catheter ablation of non-reentrant VA in patients with SHD appears good and is comparable to the outcome in patients without SHD. A slightly higher postprocedural PVC burden in patients with SHD goes along with the need of repeat ablation procedures in some patients, especially in those with dilated cardiomyopathy.

## Data Availability

The datasets used and/or analysed during the current study are available from the corresponding author on reasonable request.

## Abbreviations

DCM: dilative cardiomyopathy
ECG: electrocardiogram
HCM: hypertrophic cardiomyopathy
ICD: implanted cardioverter defibrillator
ICM: ischemic cardiomyopathy
LVEF: left ventricular ejection fraction
PVC: premature ventricular contraction
SHD: structural heart disease
VA: ventricular arrhythmias
VT: ventricular tachycardia
